# Trends in clinical stage distribution and screening detection of cancer in Osaka, Japan: Stomach, colorectum, lung, breast and cervix

**DOI:** 10.1371/journal.pone.0244644

**Published:** 2020-12-31

**Authors:** Yasuhiro Toyoda, Takahiro Tabuchi, Hitomi Hama, Toshitaka Morishima, Isao Miyashiro

**Affiliations:** 1 Department of Breast Surgery, Minoh City Hospital, Minoh, Osaka, Japan; 2 Cancer Control Center, Osaka International Cancer Institute, Osaka, Osaka, Japan; CNR, ITALY

## Abstract

We examined clinical stage distribution and proportion of screen-detected cases of stomach, colorectal, lung, female breast and cervical cancer by sex and age group using Osaka Cancer Registry data from 2000–2014. The proportion of local or in situ stage cancer had increased for all age groups in all sites, except stomach cancer in the 0–49 years group and female breast cancer in the 80 years and older group. The proportion of screen-detected cases had increased during the study period for all age groups in all cancer sites. While the proportion increased noticeably in the younger groups, there was only a slight increase in the older groups. Regarding stomach, colorectal and lung cancers, the proportion of local and in situ stage had similarly increased in the 65–79 years and 80 years and older age groups compared with younger groups, despite lower exposure to cancer screening. Regarding breast and cervical cancers, the increases in local and in situ cancer paralleled the increase in screen-detected cases. These findings suggest that the increases in early stage stomach, colorectal and lung cancers might be due not only to the expansion of screening programs but also the development of clinical diagnostic imaging or other reasons. The increases in local and in situ stage breast and cervical cancers seemed to be due to the expansion of screening. Continued monitoring of trends in cancer incidence by clinical stage may be helpful for estimating the effectiveness of screening.

## Introduction

In Japan, population-based screening for stomach and cervical cancer was introduced in accordance with the Health and Medical Service Law for the Elderly, in 1982. Screening for lung and breast cancer was subsequently added in 1988, followed by colorectal cancer screening in 1992. The modality for breast cancer screening was initially only palpation, but mammography was introduced in 2003. At present, the Ministry of Health, Labour and Welfare (MHLW) recommends the following medical tests as population-based cancer screening: biennial stomach cancer screening using upper gastrointestinal series or esophagogastroduodenoscopy (EGD) for people aged 50 years and older, annual colorectal cancer screening using fecal occult blood testing for people aged 40 years and older, annual lung cancer screening using chest X-ray for people aged 40 years and older and sputum cytology for smokers with a Brinkman Index of >600, biennial breast cancer screening using mammography for women of 40 years and older and biennial cervical cancer screening using cervical cytology for women of 20 years and older.

Several kinds of medical tests other than those recommended by MHLW have been seen in Japan as opportunistic cancer screening has increased. These include: colorectal cancer screening using colonoscopy, lung cancer screening using chest computed tomography, and breast cancer screening using ultrasonography. Regarding cancer screening rates including both population-based and opportunistic screening, the Comprehensive Survey of Living Conditions (CSLC) (a nationwide, population-based cross-sectional survey) reported that participation rates for cancer screening in 2016 were 40.9% for stomach cancer, 41.4% for colorectal cancer, 46.2% for lung cancer, 44.9% for breast cancer and 42.3% for cervical cancer [1].

The aim of population-based cancer screening is to reduce overall mortality by reducing cancer mortality. When a screening program is successful, the cancer mortality rate and the incidence rate of late stage cancer decrease [2, 3]. However, in Japan, no study has examined the effect of cancer screening on trends in cancer incidence according to clinical stage. In the present study, we examined the trends in clinical stage distribution and the proportion of screen-detected cases of stomach, colorectal, lung, female breast and cervical cancers, using population-based cancer registry data.

## Material and methods

The Osaka Cancer Registry (OCR), which was established in 1962, is a population-based cancer registry that covers the Osaka prefecture (population: 8.8 million [2015 census]) [4]. We calculated the clinical stage distribution and the proportion of screen-detected cases according to sex, age group and incident year among cancer cases using OCR data on stomach, colorectum, lung, female breast and cervical cancer incidence (International Classification of Diseases, 10^th^ revision (ICD-10); C16, C18, C19, C20, C33, C34, C50, C53, D01, D02, D05 and D06) in patients who were diagnosed between 2000 and 2014. Cases with missing sex, age and/or clinical stage were excluded from our analysis. Cases detected by autopsy were also excluded from the analysis.

Clinical stage was categorized as in situ, local, regional, and distant. In population-based cancer registry databases in Japan, cases of in situ stage stomach cancer (ICD-10; D00) were included in local stage stomach cancer (ICD-10; C16) [5].

In the OCR data, there is no distinction between population-based screening and opportunistic screening, both of which are registered as screen-detected cases.

Cases with missing detection status were regarded as non-screen-detected cases. Incident years were divided into 3-year time periods: i.e., 2000–2002, 2003–2005 up to 2012–2014. Age group was categorized as 0–49 years, 50–64 years, 65–79 years and 80 years and older. The statistical software package SAS ver.9.4 was used for the data analysis. We obtained ethical approval from the Institutional Review Board of Osaka International Cancer Institute (approval number: 18–0018) before initiating the study. The data had been anonymized before analysis.

## Results

A total of 397,715 cases of stomach, colorectal, lung, female breast and cervical cancer were identified from the OCR between 2000 and 2014. Of these, 131 cases detected by autopsy, 34 cases with missing age or sex, and 70,148 cases with unknown clinical stage were excluded. The remaining 327,402 cases were used in this study.

**Fig 1:**
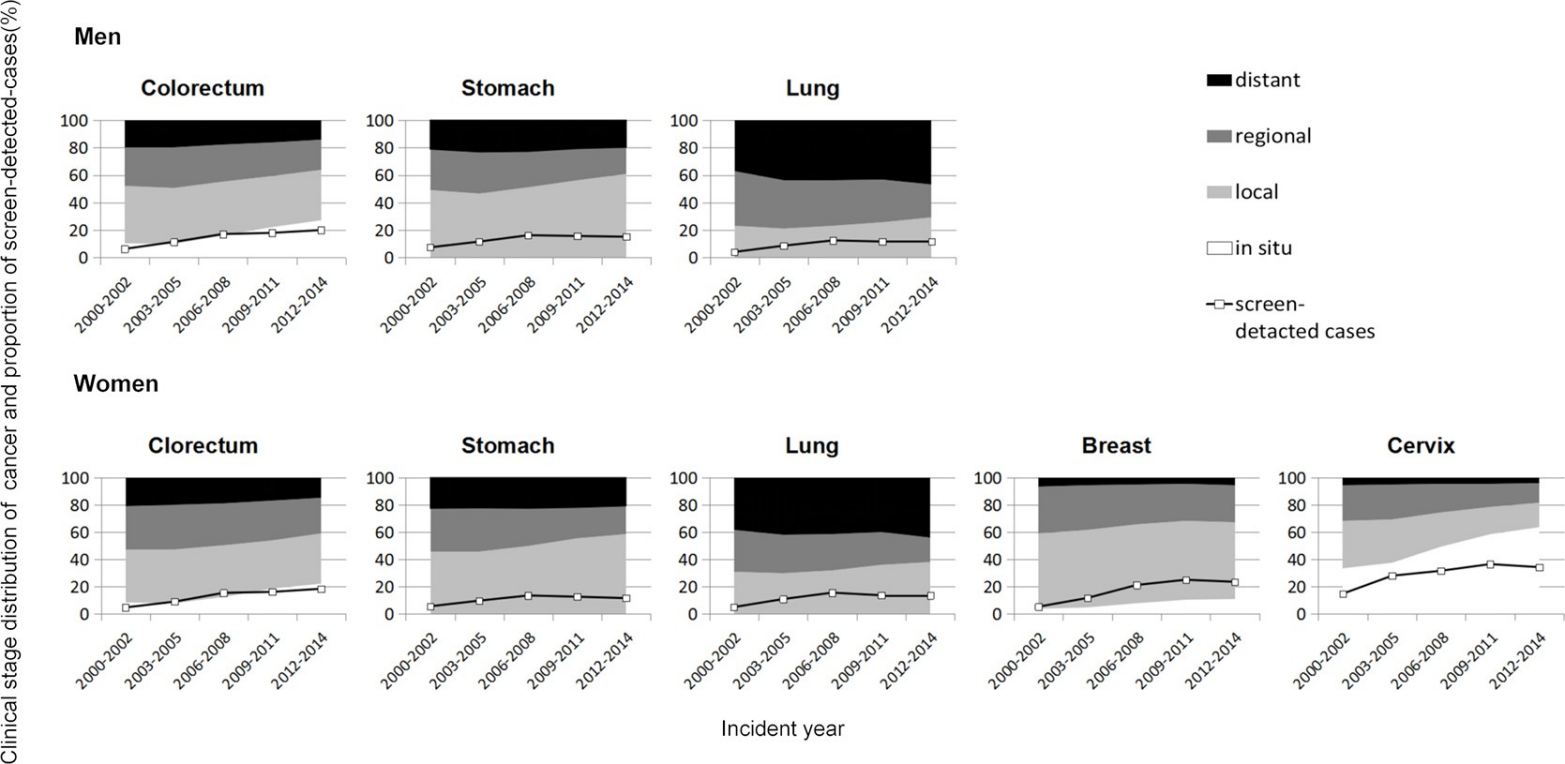
Trends in distribution of clinical stage and the proportion of screen-detected cancer cases for each 3-year period.

Fig 1 shows trends in the clinical stage distribution and the proportion of screen-detected cancer cases. The numerical values are shown in S1 Table. The proportion of local and in situ stage had increased for all cancer sites and both sexes: from 49.3% and 45.6% in 2000–2002 to 60.7% and 58.4% in 2012–2014 among men and women (respectively) for stomach cancer, from 52.2% and 47.1% in 2000–2002 to 63.8% and 59.0% in 2012–2014 among men and women for colorectal cancer, from 22.9% and 30.9% in 2000–2002 to 29.1% and 38.0% in 2012–2014 among men and women for lung cancer, from 58.8% in 2000–2002 to 67.2% in 2012–2014 for female breast cancer and from 68.5% in 2000–2002 to 81.7% in 2012–2014 for cervical cancer. The proportion of local and in situ stage lung cancer was lower than the other cancers, despite an increasing tendency in these stages. On the other hand, an increasing tendency was observed in distant stage lung cancer.

The proportion of screen-detected cancer increased for all cancer sites: from 4.1% and 5.5% in 2000–2002 to 11.8% and 11.4% in 2012–2014 among men and women (respectively) for stomach cancer, from 6.3% and 4.6% in 2000–2002 to 20.0% and 18.3% in 2012–2014 among men and women for colorectal cancer, from 4.1% and 4.9% in 2000–2002 to 11.8% and 13.4% in 2012–2014 among men and women for lung cancer, from 5.2% in 2000–2002 to 23.4% in 2012–2014 for female breast cancer and from 14.7% in 2000–2002 to 34.2% in 2012–2014 for cervical cancer.

**Fig 2:**
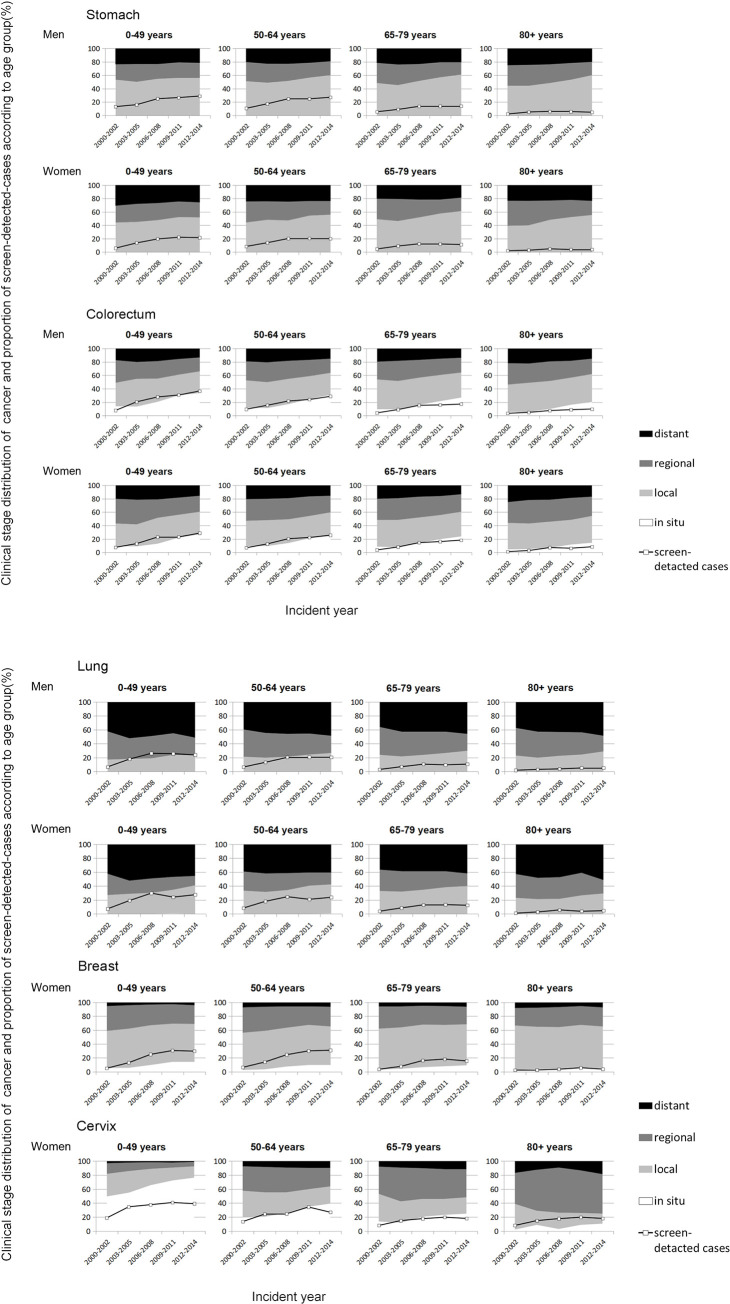
a. Trends in the age-specific distribution of clinical stage and the proportion of screen-detected cancer cases for each 3-year period. b. Trends in the age-specific distribution of clinical stage and the proportion of screen-detected cancer cases for each 3-year period.

Fig 2A and 2B show trends in the age-specific clinical stage distribution and the proportion of screen-detected cancer cases. The numerical values are shown in S2–S6 Tables. The increasing tendencies in screen-detected case were more noticeable in the younger age groups than the elderly groups for all cancer sites. The increasing tendencies in local and in situ stage cancer were confirmed for most cancer and age and sex combinations except for breast cancer in the 80 years and older group. The tendency in cervical cancer for the 80 years and older group was unclear due to the low incidence level.

Among breast cancer patients aged 80 years and older, the proportion of local and in situ stage and that of screen-detected cancer had leveled off. An increase was observed in distant stage lung cancer among men aged 50 years and older and women aged 65 years and older and in cervical cancer in women aged 50 years and older.

## Discussion

We reported simple trend statistics of cancer incidence, proportion of screen-detected cancer cases and clinical stage distribution of cancer in Osaka, Japan. As a result, an increasing tendency in the local and in situ stage was confirmed for stomach, colorectal, lung, female breast and cervical cancers. Similarly, screen-detected cases had increased, especially for the younger age groups. According to the CSLC, cancer screening rates, including opportunistic screening, among men and women aged 40 years and older in Osaka prefecture in 2013 were 32.5% and 24.0% for stomach cancer, 30.6% and 25.7% for colorectal cancer, and 24.1% and 24.9% for lung cancer. The breast cancer screening rate among women aged 40 years and older and cervical cancer screening rate among women aged 20 years and older were 21.1% and 22.8%, respectively [1].

Along with the expansion of cancer screening programs, screening-detected cancer cases had increased. When a population-based cancer screening program is successful, the cancer mortality rate and the incidence rate of late clinical stage cancer decrease [2, 3]; as the proportion of early stage cancer increases [2]. The findings of the present study seem to be almost in line with this theoretical model. The increase in screen-detected cases was parallel to the increase in the local and in situ stage, particularly in breast and cervical cancer,

Regarding stomach, colorectal and lung cancer, the increases in local and in situ stage cancer were confirmed in the elderly, although they have less exposure to cancer screening. This would suggest that screening was not the only reason for the increase in early stage cancer cases. One reason is that the elderly have more opportunities to undergo medical tests other than screening: e.g., EGD, colonoscopy and chest computed tomography. According to the CSLC in 2016 [6], the proportion of people who go to hospital for illness is higher among the elderly; the proportions for 40–49 years, 50–59 years, 60–69 years, 70–79 years and 80years and older were 27.6%, 41.9%, 58.2%, 70.8% and 73.0%, respectively. The technical development of clinical imaging might contribute to early detection of these cancers for the elderly.

An increase in the distant stage for lung cancer was observed in men aged 50 years and older and women aged 65 years and older and cervical cancer in women aged 50 years and older. Although these increases in the distant stage contradicted the increase in screen-detected cases, this might be due to improvements in OCR accuracy. The proportions of stage unknown in 2000–2002 and 2012–2014 were 29.2% and 10.9% for stomach cancer, 25.3% and 9.2% for colorectal cancer, 40.0% and 16.4% for lung cancer, 16.5% and 7.0% for female breast cancer and 13.7% and 3.1% for cervical cancer. Although the proportions had generally decreased, that of lung cancer remained relatively high. A possible reason for this might be that the development of diagnostic imaging, which enabled the detection of distant metastasis, had previously been overlooked.

The present study has some limitations. First, there were some missing cases in the OCR. Although the proportion of death certificate only (DCO) in the OCR decreased from 24.3% in 2000 to 6.2% in 2013 [7, 8], cancer incidence may be under-estimated as a whole, especially for the 2000s. Second, cases with missing detection status were included in ‘other’ in the OCR data. Although we assumed cases with missing detection status as non-screen-detected cases, the proportion of screen-detected cases might be underestimated, especially for the 2000s. Third, in order to estimate the effect of cancer screening, examining cancer incidence rate by clinical stage may be better than examining stage distribution. However, there were many cases with unknown clinical stage. Most of these cases were DCO. The proportion of cases with unknown clinical stage in the OCR data had decreased during the study period: from 28.0% in 2000–2002 to 10.5% in 2012–2014. However, the proportion for lung cancer was still high: 15.5% among men and 18.2% among women in 2012–2014. Furthermore, the proportion of unknown stage for the group aged 80 years and older remains high: from 52.3%, 46.6%, 59.9%, 33.9% and 31.8% for stomach cancer, colorectal cancer, lung cancer, female breast cancer and cervical cancer in 2000–2002 to 23.4%, 20.4%, 35.5%, 18.4% and 20.3% in 2012–2014, respectively. Therefore, we alternatively evaluated the trends with stage distribution.

In conclusion, we reported trends in clinical stage distribution and screen-detection of stomach, colorectal, lung, female breast and cervical cancers. The proportion of early stage cancer had increased, along with the expansion of cancer screening programs for breast cancer and cervical cancer in the younger age groups. Despite less exposure to cancer screening, increases in early clinical stage cancer in the elderly were also confirmed for stomach, colorectal and lung cancer. These findings suggest that the increases of early stage cancer for stomach, colorectal and lung cancer were due not only to the expansion of screening but also the development of clinical diagnostic imaging. We need to continue to monitor trends in cancer incidence by clinical stage in order to examine the effectiveness of screening.

## Supporting information

S1 Table Trends in the clinical stage distribution and the proportion of screen-detected cancer cases. (XLSX)

S2 Table Trends in the clinical stage distribution and the proportion of screen-detected cancer cases. (XLSX)

S3 Table Trends in the age-specific clinical stage distribution and the proportion of screen-detected cancer cases for colorectal cancer. (XLSX)

S4 Table Trends in the age-specific clinical stage distribution and the proportion of screen-detected cancer cases for lung cancer. (XLSX)

S5 Table Trends in the age-specific clinical stage distribution and the proportion of screen-detected cancer cases for female breast cancer. (XLSX)

S6 Table Trends in the age-specific clinical stage distribution and the proportion of screen-detected cancer cases for cervical cancer. (XLSX)

## References

[1] Cancer Registry and Statistics. Cancer Information Service, National Cancer Center, Japan [Internet]. Tokyo: Cancer Screening Rate. [Cited 20 Apr 2017]. Available from: http://ganjoho.jp/reg_stat/statistics/dl/index.html#pref_screening (in Japanese)

[2] Day NE, Williams DR, Khaw KT. Breast cancer screening programmes: the development of a monitoring and evaluation system. Br J Cancer 1989; 59: 954–958. 10.1038/bjc.1989.203 2736233 PMC2246734

[3] Chu KC, Kramer BS, Smart CR. Analysis of the role of cancer prevention and control measures in reducing cancer mortality. J Natl Cancer Inst 1991; 83: 1636–43 10.1093/jnci/83.22.1636 1749016

[4] Toyoda Y, Tabuchi T, Nakayama T, Hojo S, Yosihoka S, Wakabayashi Y et al Trends in the clinical distribution of breast cancer in Osaka, Japan. Breast Cancer 2018; 25: 250–256 10.1007/s12282-017-0807-7 29027114

[5] Lumbert R, Guilloux A, Oshima A, Pompe-Kirn V, Bray F, Parkin M et al Incidence and mortality from stomach cancer in Japan, Slovenia and the USA. Int J Cancer 2002; 97: 811–818. 10.1002/ijc.10150 11857360

[6] Summery of the Comprehensive Survey of Living Conditions. Ministry of Health, Labor and Welfare. [Cited 20 Apr 2018]. Available from: https://www.mhlw.go.jp/toukei/saikin/hw/k-tyosa/k-tyosa16/dl/16.pdf (in Japanese)

[7] Osaka Prefectural Department of Public Health and Welfare, Osaka Medical Association, Osaka Medical Center for Cancer and Cardiovascular Disease: Annual Report of Osaka Cancer Registry No.67, Cancer Incidence and Medical Care in Osaka in 2000 and the Survival in 1997, 2004 (in Japanese)

[8] Osaka Prefectural Department of Public Health and Welfare, Osaka Medical Association, Osaka International Cancer Institute: Annual Report of Osaka Cancer Registry No.81, Cancer Incidence and Medical Care in Osaka in 2013, 2017 (in Japanese)

